# A human embryonic stem cell–based model reveals the cell of origin of FOXR2-activated CNS neuroblastoma

**DOI:** 10.1093/noajnl/vdae144

**Published:** 2024-08-12

**Authors:** Hitomi N Royston, Autumn B Hampton, Dhruv Bhagat, Evonne F Pinto, Miriam D Emerson, Kosuke Funato

**Affiliations:** Department of Biochemistry and Molecular Biology, University of Georgia, Athens, Georgia, USA; Center for Molecular Medicine, University of Georgia, Athens, Georgia, USA; Center for Molecular Medicine, University of Georgia, Athens, Georgia, USA; Center for Molecular Medicine, University of Georgia, Athens, Georgia, USA; Center for Molecular Medicine, University of Georgia, Athens, Georgia, USA; Center for Molecular Medicine, University of Georgia, Athens, Georgia, USA; Department of Biochemistry and Molecular Biology, University of Georgia, Athens, Georgia, USA; Center for Molecular Medicine, University of Georgia, Athens, Georgia, USA

**Keywords:** FOXR2-activated CNS neuroblastoma, human embryonic stem cells, tumor model

## Abstract

**Background:**

FOXR2-activated central nervous system (CNS) neuroblastoma (CNS NB-FOXR2) is a recently identified subtype of brain tumor characterized by the elevated expression of the transcription factor FOXR2 mainly due to genomic rearrangements. However, the precise pathogenic mechanisms, including the cell type of origin, remain elusive.

**Methods:**

A gene expression analysis of patient tumors was performed to identify putative cell types of origin. Based on this prediction, a new human embryonic stem cell–based model was developed to validate the origin and to examine the molecular and cellular mechanisms underlying the formation of CNS NB-FOXR2.

**Results:**

Our data showed that CNS NB-FOXR2 tumors express a high level of lineage marker genes associated with the medial ganglionic eminence (MGE), a transient structure located in the developing ventral forebrain. Our model confirmed the cell-type-specific effect of FOXR2 on the proliferation and in vivo tumorigenicity. Additionally, we found that FOXR2 overexpression activated the MEK/ERK signaling pathway through a suppression of the endogenous RAS inhibitor DIRAS3. The MEK inhibitor trametinib suppressed the proliferation of FOXR2-expressing MGE progenitors more than nonexpressing cells.

**Conclusions:**

Our study collectively demonstrates that MGE progenitors are the cell of origin of CNS NB-FOXR2 and that FOXR2 activates the MEK/ERK signaling pathway, providing a potential therapeutic target.

**Key Points**

- Medial ganglionic eminence progenitors are the cell of origin of FOXR2-activated central nervous system neuroblastoma.
- FOXR2 activates the MEK/ERK signaling pathway through a suppression of DIRAS3.

**Importance of the Study**

FOXR2-activated central nervous system neuroblastoma (CNS NB-FOXR2) is a recently recognized, rare subtype of pediatric brain tumors. Due to its small patient population, it is very challenging to obtain patient specimens and establish cell lines, which significantly limits the advance of research. Previous studies have revealed the oncogenic role of FOXR2 in various types of brain tumors; however, the precise pathogenic mechanisms underlying the formation of CNS NB-FOXR2, including the cell type of origin, are still unclear, and therapies tailored to this subtype have not been established. By utilizing a new model that more accurately recapitulates the molecular features of patient tumors, this study provides novel insights into the cellular and molecular mechanisms of tumorigenesis driven by the FOXR2 activation, paving the way for developing effective treatments for this devastating and understudied disease.

Brain tumors are a leading cause of pediatric cancer-related deaths, affecting over 5000 children annually in the United States, with a mortality of nearly 500.^1^ Recent studies have uncovered comprehensive molecular and genetic profiles of pediatric brain tumors, identifying new molecularly defined subtypes.^2^ This led to a substantial update to the World Health Organization (WHO) classification of central nervous system (CNS) tumors in 2021.^3,4^ Notably, primitive neuroectoderm tumors (PNETs), a malignant form of pediatric brain tumor, underwent significant reevaluation and reclassification into distinct subtypes. One recently identified subtype, FOXR2-activated central nervous system neuroblastoma (CNS NB-FOXR2), is characterized by genomic rearrangements at the Forkhead box gene R2 (*FOXR2*) locus. FOXR2, a member of an evolutionarily conserved transcription factor family, is specifically expressed in male reproductive organs.^5^ CNS NB-FOXR2 tumors exhibit an ectopic expression of FOXR2 due to genomic rearrangement, including the insertion of mitochondrial gene.^6–8^ Previous studies have shown the functional interaction of FOXR2 with Myc/N-Myc oncoproteins as well as with the ETS transcription factor family.^5,8,9^ Histological features of CNS NB-FOXR2 include poorly differentiated neuroblastic cells with dark nuclei and scant cytoplasm, suggesting an embryonic origin of CNS NB-FOXR2. However, the precise mechanisms underlying subtype-specific tumorigenesis, including the cell type of origin, remain elusive. One of the major challenges in studying CNS NB-FOXR2 is the small patient population. CNS NB-FOXR2 consists of less than 3% of all high-grade pediatric brain tumors,^10^ and alterations at the *FOXR2* locus are rarely examined for diagnosis. Thus, it is challenging to obtain a patient specimen and establish a cell line. As far as our knowledge goes, no patient-derived cell line has been established from CNS NB-FOXR2.

Human pluripotent stem cells, including human embryonic stem cells (hESCs) and induced pluripotent stem cells (iPSCs), are a useful platform to model various diseases, including pediatric brain tumors, as they can be differentiated into a particular cell type that is relevant to disease pathogenesis. Here, we developed a new hESC model to identify the cell type of origin and elucidate the pathogenic mechanisms of CNS NB-FOXR2. A gene expression profile analysis of the patient tumors revealed heightened expression of genes associated with the medial ganglionic eminence (MGE), a transient structure located in the developing ventral forebrain. The MGE is the major source of GABAergic inhibitory interneurons during brain development; however, the role of the MGE lineage in tumorigenesis has not been explored. To test our hypothesis that CNS NB-FOXR2 tumors originate from the MGE lineage, we overexpressed FOXR2 in hESC-derived MGE progenitors and cortical progenitors as a control cell type and investigated the cell-type-specific effects of FOXR2 on cell viability and in vivo tumorigenicity. Additionally, we analyzed the gene expression profiles of our model cells and identified a FOXR2 downstream pathway that can serve as a potential therapeutic target for CNS NB-FOXR2.

## Materials and Methods

### Cell Culture

H1 hESCs (NIHhESC-10-0043) were obtained from WiCell and cultured in Essential 8 FLEX medium (Thermo Fisher) on cell culture dishes coated with recombinant Vitronectin (Thermo Fisher). Karyotyping and mycoplasma tests were conducted annually.

### Embryonic Stem Cell Differentiation

HESCs were differentiated into MGE progenitors and cortical progenitors using a previously described protocol^11^ with minor modifications. A total of 250 000 cells were plated on a Vitronectin-coated 24-well plate. The following day (day 0), the medium was switched to Essential 6 medium (Thermo Fisher) with 200 nM of BMP inhibitor LDN-193189 (STEMCELL Technologies), 10 μM of SMAD inhibitor SB-431542 (STEMCELL Technologies), and 2 μM of Wnt inhibitor XAV-939 (Cayman Chemical). From days 6 to 10, cells were cultured in DMEM/F12 medium with N2 and B27 supplements (Thermo Fisher). On day 10, cells were dissociated with Accutase (Innovative Cell Technologies) and replated on a 12-well plate coated with poly-ornithine (MilliporeSigma), laminin (Bio-techne), and fibronectin (MilliporeSigma). To obtain MGE progenitors, cells were treated with 1 μM of Smoothened Agonist (SAG; Cayman Chemical) for 6 days to acquire the ventral identity. No SAG was added for cortical progenitors. After this patterning period, cells were dissociated, replated on poly-ornithine/laminin/fibronectin-coated plates using high-density droplets, and cultured with N2, B27, and 0.4 ng/mL of epidermal growth factor (STEMCELL Technologies) for 14 days. From day 30, cells were cultured with a neural differentiation medium, supplemented with 20 ng/mL of brain-derived neurotrophic factor (STEMCELL Technologies) and 200 μM of ascorbic acid (MilliporeSigma) for an additional 14 days.

### Immunohistochemistry

Cells were fixed with 4% paraformaldehyde, rinsed with PBS, and incubated with a blocking solution containing 10% FBS and 0.3% Triton X-100 for 1 hour. Antibodies, including rat monoclonal antibody against NKX2-1 (SP141; 1:200) from Abcam and PAX6 (AD2.35; 1:200) from BioLegend, were diluted in the blocking solution and applied to samples overnight at 4°C. Subsequently, samples were washed 3 times with PBS, incubated with secondary antibodies in PBS with 0.1% bovine serum albumin, followed by nuclear staining using DAPI. Cryosections were stained with antibodies against human nuclear antigen (Abcam; 235-1; 1:2000), Ki67 (R&D, 1297A, 1:200), human NCAM (Santa Cruz; ERIC1; 1:400), OLIG2 (Cell Signaling; E6G6Q; 1:200), and Synaptophysin (ABclonal; 1:200). TUNEL assay was performed using TUNEL Assay Kit (Abcam).

### In Silico Analysis of Putative Cell of Origin

Gene expression profile analysis was conducted as described previously.^12^ Briefly, gene expression profiles from 3 brain tumor data sets (GSE34824, GSE36245, and GSE73038) were combined and compared with a single-cell RNA-seq data set obtained from human fetal brains.^13^ Enrichment/depletion scores were calculated by a modified version of gene set enrichment analysis (GSEA) on the GenePattern server (https://cloud.genepattern.org).

### RNA-seq

Total RNA was extracted using Trizol reagent (Thermo Fisher), and subsequent library preparation and next-generation sequencing were conducted by GENEWIZ. Data processing was performed on the Galaxy public server (https://usegalaxy.org). Following the removal of low-quality reads and adaptor sequences, reads were aligned with the human genome (hg38) using HISAT2 with default parameters. Gene expression levels were quantified by featureCounts and normalized to TPM (transcripts per kilobase million). After filtering out genes with low expression or variation across samples, the expression data were scaled by *Z-*scores and underwent principal component analysis (PCA) using the R software.

### Western Blot

Cells were lysed in RIPA buffer, and after a 30-minute centrifugation at 16 900 rpm, the supernatant was collected. Protein concentration was determined using the Bradford Assay (Bio-Rad). Lysates were boiled for 5 minutes in Laemmli sample buffer, separated by electrophoresis on a 10% Bis-Tris gel in SDS running buffer for 1.5–2 hours, and then transferred to a nitrocellulose or PVDF membrane. To prevent nonspecific protein binding, the membrane was blocked with a 5% Blotting-Grade Blocker (Bio-Rad) in TBS-T. The membrane was incubated at 4°C overnight in the blocking buffer with primary antibodies. Anti-FLAG M2 mouse monoclonal antibody was obtained from MilliporeSigma, and anti-GAPDH (14C10, 1:2000), anti-phosphorylated ERK (D13.14.4E, 1:2000), and anti-total ERK (L34F12, 1:2000) were from Cell Signaling Technology, and anti-DIRAS3/ARHI was from Abcam (ab107051, 1:2000). After 4 washes with TBS-T, the blot was incubated with the respective secondary antibodies for mouse (1:5000) or rabbit (1:5000) at room temperature for 30 minutes. Clarity ECL Western Blotting Substrates (Bio-Rad) were used for detection according to the manufacturer’s instructions. For re-blotting, antibodies were stripped by Restore Western Blot Stripping Buffer (Thermo Fisher).

### Quantitative Real-Time PCR

Total RNA was extracted using Trizol. For each sample, 1 μg of total RNA was reverse-transcribed using the RevertAid RT Reverse Transcription Kit (Thermo Fisher). Amplified material was detected using SYBR Green Master Mix (Thermo Fisher) on a CFX Opus 384 Real-Time PCR Detection System (Bio-Rad). All results were normalized to a β-actin control. The sequences of the primers are shown in Supplementary Table 5.

### Lentivirus Production

Lentiviral vectors were transfected in HEK293T cells with packaging vectors in the presence of polyethylenimine (Polysciences). Viral supernatants were collected 72 hours after transfection, and viral particles were concentrated by ultracentrifugation at 49 000*g* for 1.5 hours at 4°C.

### Intracranial Transplantation

A total of 500 000 cells suspended in 2 µL of PBS were loaded into a Hamilton syringe and injected to the target coordinates (2 mm anterior and 2 mm lateral right from the bregma, 2 mm deep from the skull surface). After a brief 2-minute interval to ensure optimal positioning, the cell suspension was injected at less than 1 µL/min. To prevent any potential backflow, the needle was left in place for 5 minutes before being slowly withdrawn at a rate of 0.5 mm per minute. To maintain sterility throughout the procedure, all equipment was sterilized between animals. All animal experiments were approved by University of Georgia Institutional Animal Care and Use Committee.

### Bioluminescence Imaging

Prior to imaging, animals were anesthetized using isoflurane, and 200 µL of d-luciferin was administered via retro-orbital injection. The animals were placed in the imaging chamber of the bioluminescence imaging system, Newton 7.0 FT-500 (Vilber Lourmat). Bioluminescence signals emitted by the luciferase-expressing cells were detected at a 5-minute exposure time and quantified using Kuant software.

### Quantification and Statistical Analysis

*P* values were calculated by ANOVA and Student’s *t* tests for pairwise comparisons unless indicated otherwise. When data were not normally distributed based on the Shapiro test, *P* values were calculated by the Wilcoxon rank sum test. The numbers of biological replicates for each experiment are shown in figure legends.

## Results

### Computational Analysis Predicts CNS NB-FOXR2 Originates From the MGE Lineage

**Figure 1.**
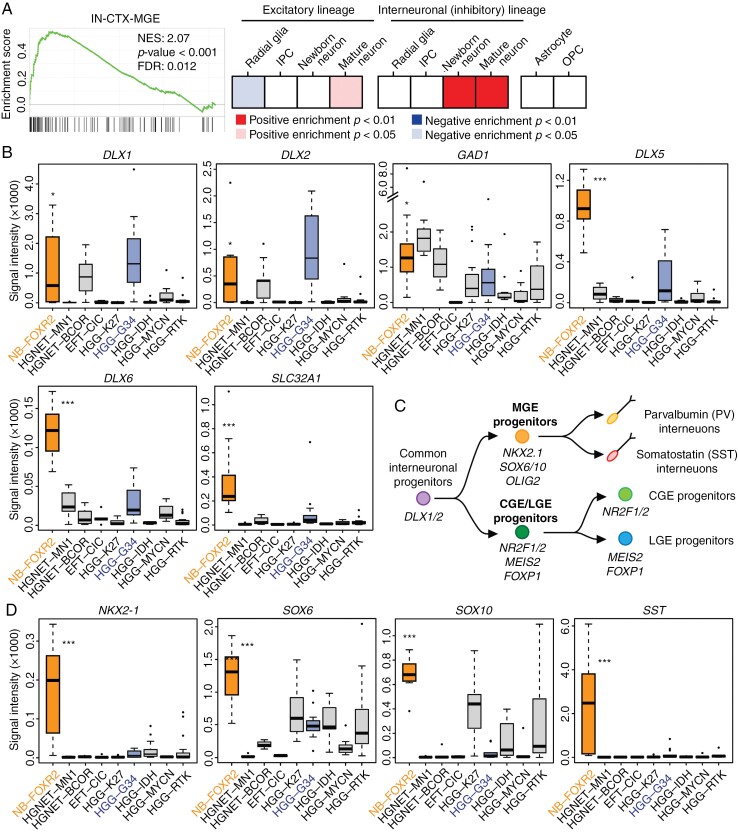
FOXR2-activated central nervous system neuroblastoma (CNS NB-FOXR2) tumors highly express medial ganglionic eminence (MGE) markers. (A) Gene set enrichment analysis (GSEA) showed enrichment of MGE-type interneuron signature in CNS NB-FOXR2. A representative GSEA plot of mature MGE-type interneurons (IN-CTX-MGE) is shown on the left, and enrichment of indicated gene expression signatures is shown on the right. FDR = false discovery rate; IPC = intermediate progenitor cells; NES = normalized enrichment score; OPC = oligodendrocyte progenitor cells. (B) Boxplots show the expression levels of interneuronal marker genes in various subtypes of pediatric brain tumors. The top, middle, and bottom of each box represent the 75, 50, and 25 percentiles, respectively. HGG = high-grade glioma; HGNET = high-grade neuroepithelial tumor. (C) Schematic illustration of interneuronal lineages and their maker genes. (D) Boxplots show the expression levels of MGE marker genes in various subtypes of pediatric brain tumors. *P < .05, ***P < .001.

To identify the putative cell type of origin, we used expression profile data sets of patient tumor samples and conducted an in silico analysis as described previously.^12^ First, we combined expression profile data from 3 pediatric brain tumor data sets (GSE34824, GSE36245, and GSE73038) and extracted samples classified into one of the following subtypes: CNS neuroblastoma with FOXR2 activation (NB-FOXR2), high-grade neuroepithelial tumor with MN1 alteration (HGNET-MN1), high-grade neuroepithelial tumor with BCOR alteration (HGNET-BCOR), CNS Ewing sarcoma family tumor with CIC Alteration (EFT-CIC), high-grade glioma with H3K27 alteration (HGG-K27), high-grade glioma with H3G34 alternation (HGG-G34), high-grade glioma with IDH alternation (HGG-IDH), high-grade glioma with MYC alternation (HGG-MYC), and high-grade glioma with receptor tyrosine kinase alternation (HGG-RTK). The final data set contains 120 samples, including 10 samples of CNS NB-FOXR2. Next, gene expression signatures representing distinct cell populations in the developing human brain are extracted from single-cell RNA-seq data.^13^ The GSEA was used to calculate the enrichment or depletion of these signatures in CNS NB-FOXR2 compared to the other subtypes of brain tumors in children and young adults. Notably, our data showed a significant enrichment of signatures associated with interneurons in CNS NB-FOXR2 (Figure 1A). CNS NB-FOXR2 exhibits an elevated expression of interneuronal marker genes, including DLX transcription factor genes and *GAD1*, which encodes an enzyme responsible for glutamate-to-GABA conversion (Figure 1B). Consistent with previous studies, HGG-G34 subtype tumors that harbor histone H3.3G34R/V mutation also highly express these interneuronal marker genes.^12,14^ During the maturation of interneurons, the expression of DLX1 and DLX2 decreases, and that of DLX5 and DLX6 increases reciprocally.^15^ Compared to HGG-G34, CNS NB-FOXR2 shows a lower expression of *DLX1* and *DLX2* and a higher expression of *DLX5* and *DLX6*. Additionally, CNS NB-FOXR2 expresses a higher level of *SLC32A1* that encodes the Vesicular GABA Transporter, an enzyme required for GABA secretion from mature interneurons. This expression pattern and the GSEA analysis collectively indicate the presence of mature interneurons in CNS NB-FOXR2. Corroborating this finding, the synaptic vesicle protein synaptophysin is highly expressed and thus used as a molecular marker for the diagnosis of CNS NB-FOXR2.^16–18^ During the fetal brain development, interneurons are predominantly generated in the ganglionic eminence, a transient structure located in the ventral forebrain.^19^ The ganglionic eminence is composed of 3 areas: MGE, caudal ganglionic eminence (CGE), and lateral ganglionic eminence (LGE). A recent single-cell RNA-seq study indicates that the interneuronal lineage first diverges into MGE progenitors and CGE/LGE common progenitors^20^ (Figure 1C). MGE marker genes *NKX2.1*, *SOX6*, and *SOX10* are highly expressed in CNS NB-FOXR2, while the expression of CGE/LGE marker genes, including *NR2F2* (also known as COUP-TFII), *MEIS2*, and *FOXP1*, is relatively low compared to the other subtypes (Figure 1D; Supplementary Figure 1A). The MGE mainly produces 2 groups of interneurons defined by the expression of somatostatin (SST or SOM) and parvalbumin, respectively. The *SST* gene, which encodes somatostatin, is almost exclusively expressed in CNS NB-FOXR2, further indicating that CNS NB-FOXR2 originates from the MGE lineage. Another feature of CNS NB-FOXR2 was the heightened expression of OLIG2, which is also highly expressed in the MGE (Supplementary Figure 1B).^21^ Corroborating this, analysis of a single-cell RNA-seq data set from human fetal brains demonstrated co-expression of *OLIG2* and *NKX2.1* in a subset of interneuronal cells (Supplementary Figure 1C). Taken together, these data indicate that CNS NB-FOXR2 likely originates from the MGE lineage.

### CNS NB-FOXR2 Model Exhibits Robust Proliferation in MGE Progenitors Upon FOXR2 Overexpression

**Figure 2.**
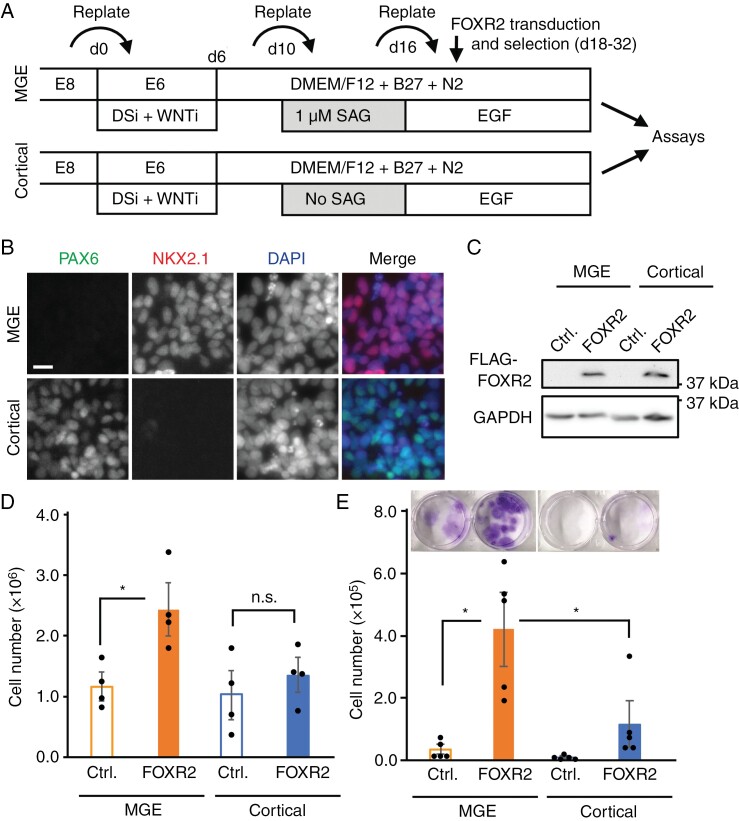
The human embryonic stem cell (hESC)–based model shows the cell-type-specific effect of FOXR2. (A) Schematic illustration of the protocols to differentiate hESCs from medial ganglionic eminence (MGE) progenitors and cortical progenitors, respectively. DSi = Dual Smad inhibition (SB-431542 and LDN-193189); WNTi = Wnt inhibitor (XAV-939). (B) Immunostaining showed the specific expression of cortical and MGE markers. Cells were stained with antibodies against PAX6 (cortical progenitor marker) and NKX2.1 (MGE progenitor markers) and counterstained with DAPI. Scale bar: 20 μm. (C) Western blotting showed the expression of FOXR2. (D) The effect of FOXR2 overexpression on the viability of cortical progenitors and MGE progenitors. 500 000 cells/well were plated on a 24-well plate. After 6 days of incubation, the number of live cells was counted by trypan-blue staining. (E) The effect of FOXR2 overexpression under low-density conditions. 6000 cells/well were plated on a 24-well plate. After 6 days of incubation, the number of live cells was counted by trypan-blue staining. Crystal violet staining is shown on the top. Bars in (D) and (E) represent mean ± SEM (*n* = 3–5). *P < .05, n.s. = not significant.

To experimentally confirm the prediction that CNS NB-FOXR2 originates from MGE-type interneuronal cells, we established a novel hESC-based tumor model tailored explicitly for CNS NB-FOXR2. We optimized a previously published chemically defined differentiation protocol^7^ and differentiated hESC into MGE progenitors (Figure 2A). In addition, as a control cell type, we differentiated hESC to cortical progenitors, which are localized in the dorsal forebrain and give rise mostly to excitatory neurons. Proper differentiation and regional patterning of the cells were confirmed by the expression of the MGE marker NKX2.1 and the dorsal progenitor marker PAX6 by immunostaining (Figure 2B). Given that CNS NB-FOXR2 tumors are characterized by elevated FOXR2 expression, the wild-type *FOXR2* gene was lentivirally transduced into MGE progenitors and cortical progenitors and expressed under the constitutively active human PGK1 promoter (Figure 2C). FOXR2 overexpression led to a statistically significant increase in the proliferation of MGE progenitors but not that of cortical progenitors (Figure 2D). Furthermore, in a low-density culture condition where normal neural progenitors typically struggle to survive and proliferate, MGE progenitors exhibited robust viability upon FOXR2 overexpression, whereas cortical progenitors demonstrated considerably lower viability (Figure 2E). These data collectively indicate the cell-type-specific effects of FOXR2 and support our hypothesis that CNS NB-FOXR2 is derived from the MGE lineage.

### FOXR2 Expressing MGE Progenitors Form Tumors In Vivo

**Figure 3.**
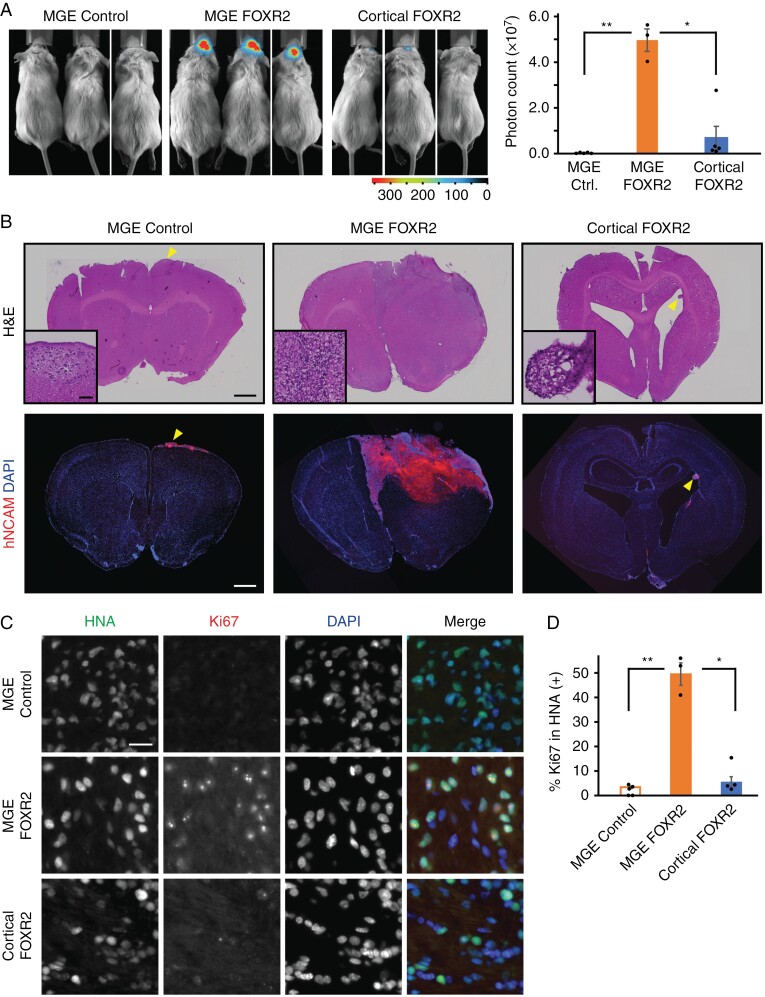
FOXR2-expressing medial ganglionic eminence (MGE) progenitors form tumors in vivo. (A) Bioluminescence imaging from the indicated animals 6 months after transplantation. Quantification of photon counts is shown on the right. (B) Representative images of cryosections stained with hematoxylin and eosin or anti-human NCAM antibody and DAPI. Images are composites of 2–4 images. Arrowheads show grafts. Scale bar: 1 mm. High-magnification images are shown in the insets (scale bar: 50 μm). (C) Cryosections from the indicated animals were immunostained for human-specific antigen (HNA) and Ki67 and counterstained with DAPI. Scale bar: 20 μm. (D) Quantification of immunostaining for HNA and Ki67. The percentage of Ki67-positive cells in HNA-positive cells was calculated. Bars in (A) and (D) represent mean ± SEM (*n* = 3–5). **P < .01.

Next, we examined in vivo tumorigenicity by genetically labeling the cells with firefly luciferase and transplanting them into the brains of the immunodeficient NSG mice. Bioluminescence imaging showed that mice bearing FOXR2-expressing MGE cells (hereafter referred to as MGE FOXR2 mice) exhibited significantly stronger signals compared to those with control MGE cells (MGE control mice) and FOXR2-expressing cortical progenitors (cortical FOXR2 mice), further confirming the cell-type-specific role of FOXR2 in tumorigenesis (Figure 3A). Six months after the transplantation, 2 out of 3 MGE FOXR2 mice reached the humane endpoint, while animals bearing either control MGE cells or FOXR2-expressing cortical progenitors did not show any symptoms (Supplementary Figure 2A). To characterize the grafts, cryosections of the brains were analyzed using hematoxylin and eosin (H&E) staining as well as immunostaining. H&E staining and anti-human NCAM staining showed the formation of large tumors in MGE FOXR2 mice (Figure 3B). On the other hand, control MGE mice and cortical FOXR2 mice harbored much smaller grafts, mainly around the outer layer of the cortex or the ventricles (Figure 3B, arrowheads). Consistent with the in vitro data shown in Figure 2, Ki67 staining indicated a significantly higher in vivo proliferation in MGE FOXR2 mice compared to control MGE mice and cortical FOXR2 mice (Figure 3C). Additionally, immunostaining showed that tumors in MGE FOXR2 mice exhibited the expression of CNS NB-FOXR2 markers, including synaptophysin, NKX2.1, SOX6, SOX10, and OLIG2, indicating that our model molecularly resembles patient tumors (Supplementary Figure 2B).

### The MEK/ERK Signaling Pathway Is Activated by FOXR2 Overexpression

**Figure 4.**
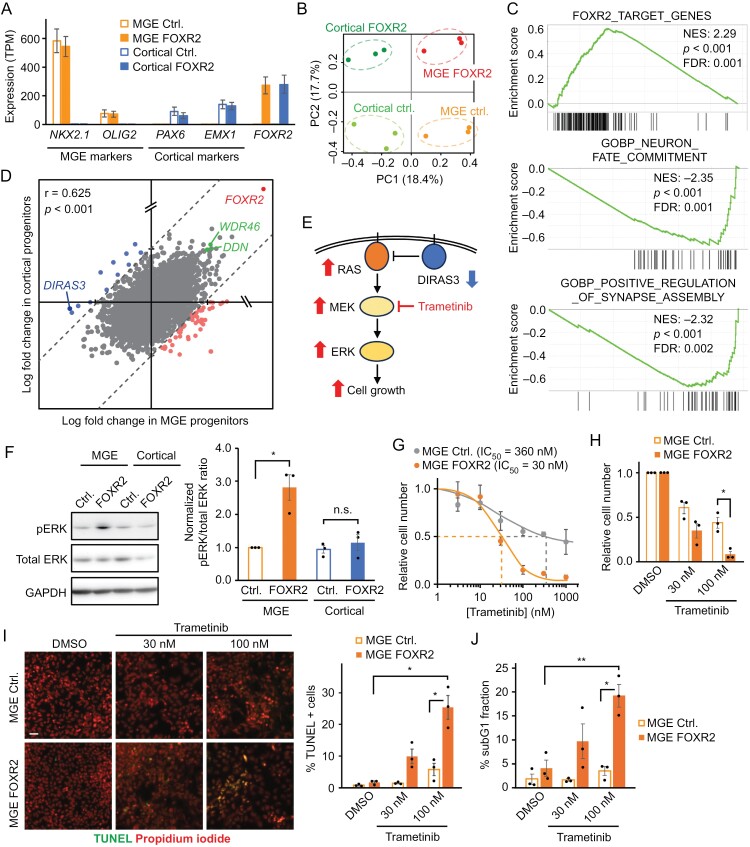
FOXR2 enhances the MEK/ERK signaling pathway through a suppression of DIRAS3. (A) RNA-seq showed the expression of lineage marker genes and FOXR2. (B) Principal component analysis showed changes in expression profiles by FOXR2 overexpression. (C) Gene set enrichment analysis plots show positive or negative enrichment of the indicated gene signatures in FOXR2-expressing medial ganglionic eminence (MGE) progenitors compared to control MGE progenitors. (D) Changes in gene expression by FOXR2 overexpression in MGE progenitors (*x*-axis) and cortical progenitors (*y*-axis) are shown. Each dot represents a gene. (E) Schematic illustration of the suppressive role of DIRAS3 on the MEK/ERK signaling pathway. (F) Western blotting showed an elevated level of ERK phosphorylation in FOXR2-expressing MGE progenitors. Quantification of band intensities is shown on the right. (G, H) FOXR2-expressing MGE progenitors were more sensitive to the MEK inhibitor trametinib compared to control MGE progenitors. Cells were treated with either DMSO or trametinib for 6 days, and the number of live cells was counted by trypan-blue staining. (I, J) Trametinib treatment induced cell death in FOXR2-expressing MGE progenitors in a dose-dependent manner. Cells were treated with either DMSO or trametinib for 4 days and subjected to TUNEL assay (I) and subG1 assay (J). Scale bar in (I): 20 μm. Bars in (A, E, F, H, I, and J) represent mean ± SEM (*n* = 3–5). *P < .05, **P < .01, n.s. = not significant.

To understand the molecular mechanism driving the formation of CNS NB-FOXR2, we conducted an RNA-seq analysis to capture gene expression profiles from 4 samples: control MGE progenitors, FOXR2-expressing MGE progenitors, control cortical progenitors, and FOXR2-expressing cortical progenitors. Our RNA-seq data confirmed the specific expression of marker genes for MGE progenitors (such as *NKX2.1* and *OLIG2*) and cortical progenitors (such as *PAX6* and *EMX1*) in their corresponding cell types (Figure 4A). PCA shows remarkable changes in expression profiles in both MGE progenitors and cortical progenitors (Figure 4B). Consistent with the previous study,^5^ GSEA showed that FOXR2 overexpression resulted in a statistically significant upregulation of known FOXR2 target genes (derived from ChIP-seq data of the MDA-MB-231 breast adenocarcinoma cell line)^9^ (Figure 4C; Supplementary Table 2). Additionally, GSEA showed a significant downregulation of gene sets associated with neuronal differentiation. Upregulated genes include *WDR46* and *DDN*, both of which are highly and specifically expressed in CNS NB-FOXR2 patient tumor tissues compared to the other subtypes (Figure 4D; Supplementary Figure 3A). Although the transcriptional impact of FOXR2 overexpression in cortical progenitors was similar to that in MGE progenitors (*r* = 0.625, *P* < .001), we identified differentially regulated genes between these 2 cell types (Supplementary Table 3). Among them, *DIRAS3* exhibited a significant downregulation upon FOXR2 overexpression in MGE progenitors but no effect in cortical progenitors (Supplementary Figure 3B). *DIRAS3* (also known as *ARHI* or *NOEY2*) encodes DIRAS family GTPase 3 that directly binds RAS oncoprotein and inhibits the MEK/ERK signaling pathway^22,23^ (Figure 4E). Western blotting confirmed that FOXR2 overexpression resulted in an increased level of ERK phosphorylation in MGE progenitors but not in cortical progenitors (Figure 4F). The MEK/ERK signaling pathway regulates gene expression via transcription factors including ELK1, GABP, and CREB. GSEA using the patient expression profile data showed a significant enrichment of ELK1, GABP, and CREB signatures in CNS NB-FOXR2 compared to the other subtypes of pediatric brain tumors (Supplementary Figure 3D; Supplementary Table 4). To validate the role of the elevated MEK/ERK signaling pathway in the survival and proliferation of CNS NB-FOXR2 cells, FOXR2-expressing MGE progenitors and control MGE progenitors were treated with the FDA-approved MEK inhibitor trametinib (brand name Mekinist). Trametinib treatment significantly suppressed the viability of FOXR2-expressing MGE progenitors in a much lower concentration (IC_50_ = 30 nM) compared to control MGE progenitors (IC_50_ = 360 nM) (Figure 4G and H). Additionally, both TUNEL and subG1 assays showed that trametinib treatment significantly induced cell death in FOXR2-expressing MGE progenitors but not in control MGE progenitors (Figure 4I and J; Supplementary Figure 3E). These observations collectively indicate that DIRAS3 is downregulated by FOXR2 and acts as a tumor suppressor by activating the MEK/ERK signaling pathway.

## Discussion

CNS NB-FOXR2 was previously classified under CNS-primitive neuroectodermal tumor (CNS-PNET). A comprehensive analysis of DNA methylation and transcriptomics in a substantial CNS-PNET cohort by Sturm et al. identified 4 distinct CNS tumor subtypes, each linked to recurrent genetic events and named accordingly.^2^ In the “2021 WHO Classification of Tumors of the Central Nervous System,” CNS NB-FOXR2 emerged as a novel malignant subtype. Our data strongly indicate that ventral forebrain interneuronal progenitors residing in the MGE serve as the cell type of origin for this subtype, supporting the notion of early tumor initiation during fetal brain development.

Our model serves as a valuable platform for dissecting the mechanistic role of FOXR2 in a particular cellular context. Previous models neither overexpressed FOXR2 specifically into the MGE lineage nor confirmed the expression of CNS NB-FOXR2 marker genes. The unique expression profile of CNS NB-FOXR2, featured by the expression of the MGE markers, strongly indicates the interaction between FOXR2 and molecules specifically expressed and/or regulated in the MGE lineage. Our study identified that FOXR2 activated the MEK/ERK signaling pathway, specifically in the MGE progenitors. However, further studies are essential to clarify the molecular mechanisms underlying the cell-type-specific effects of FOXR2. Importantly, our study shows that FOXR2 overexpression alone is sufficient to induce oncogenic transformation in MGE progenitors (Figure 3). This is contrary to a previous mouse study that showed that the inactivation of p53 is required for tumorigenicity induced by Foxr2 overexpression.^24^ It is worth mentioning that p53 mutation has not been reported in CNS NB-FOXR2; however, other mechanisms to suppress the p53 pathway, such as activation of MDM family genes, have not been ruled out.

Beyond CNS NB-FOXR2, this model can be readily expanded to explore other brain tumor subtypes associated with FOXR2 alternations. FOXR2 gene aberrations are noted in some medulloblastoma cases.^25^ Another subtype of pediatric brain tumor with FOXR2 overexpression is pineoblastoma (PB). PB-MYC/FOXR2 is characterized by MYC amplification and overexpression of FOXR2. FOXR2 activation is also observed in a subset of diffuse midline glioma.^5^ These FOXR2-related neoplastic entities involve multiple oncogenes, some regulated by FOXR2 and others possibly influencing FOXR2. Identifying these factors in the FOXR2 network offers potential avenues for more precise targeted therapies, enhancing outcomes in pediatric brain tumors.

Our data showed that the MEK/ERK signaling pathway is upregulated by FOXR2 through suppression of the endogenous RAS inhibitor DIRAS3 and thus can be a potential therapeutic target for CNS NB-FOXR2 (Figure 4). Trametinib is a potent, blood–brain barrier-permeable inhibitor for MEK1 and MEK2. Trametinib is approved by the FDA for the treatment of BRAFV600E mutated metastatic melanoma as well as BRAFV600E mutated low-grade glioma (in combination with dabrafenib). Additionally, trametinib and other MEK inhibitors are currently tested in multi-institutional clinical trials for pediatric brain tumors. This study provides useful knowledge to help interpret the outcome of these clinical trials as well as to design future clinical trials, further iterating the importance of the genetic and molecular stratification of pediatric brain tumor patients.

Taken together, this study offers novel insight into the cellular and molecular mechanisms of tumorigenesis driven by the FOXR2 activation, paving the way for developing effective treatments for this devastating disease.

## Supplementary Material

vdae144_suppl_Supplementary_Data

## Data Availability

RNA-seq data were deposited into sequence read archive database under accession number PRJNA1099152.
