# Supplementary figures and images for: A human embryonic stem cell–based model reveals the cell of origin of FOXR2-activated CNS neuroblastoma
Source: Neurooncol Adv. 2024 Aug 12;6(1):vdae144. doi: 10.1093/noajnl/vdae144 (PMC11364937; doi:10.1093/noajnl/vdae144)

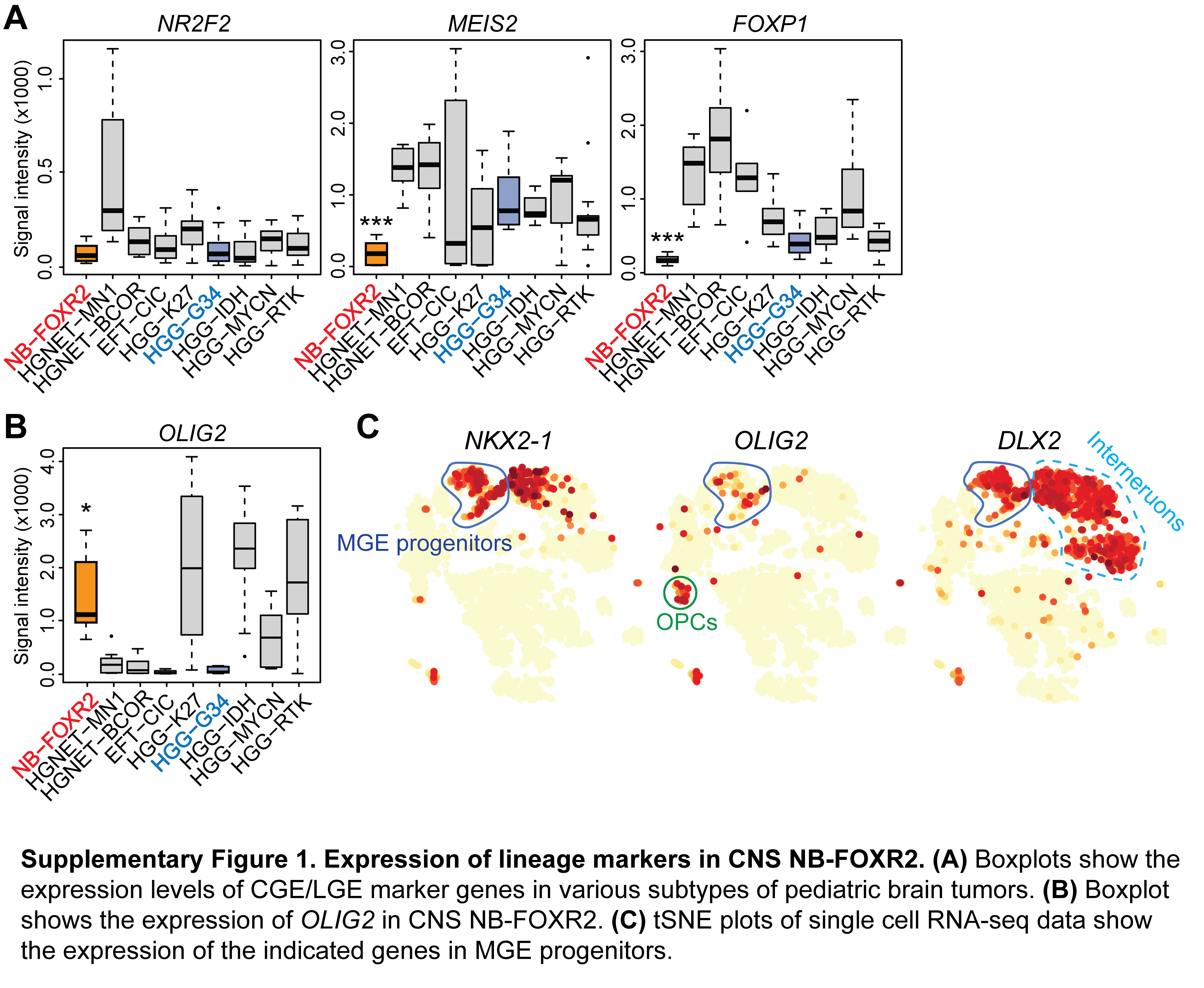

Supplement: vdae144_suppl_Supplementary_Data [file vdae144_suppl_supplementary_data.zip › Royston_et_al_FOXR2_FigS1c.tif]

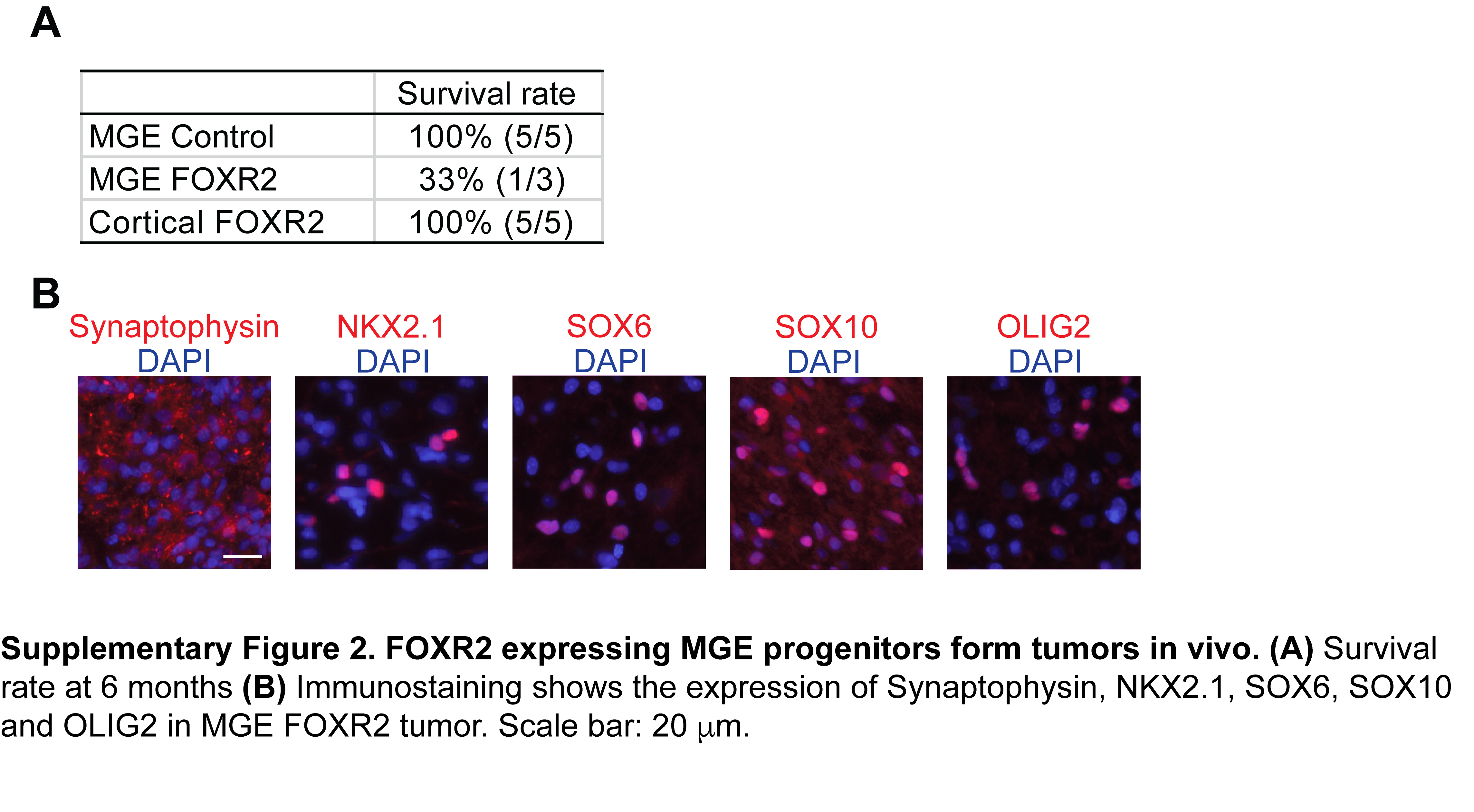

Supplement: vdae144_suppl_Supplementary_Data [file vdae144_suppl_supplementary_data.zip › Royston_et_al_FOXR2_FigS2c.tif]

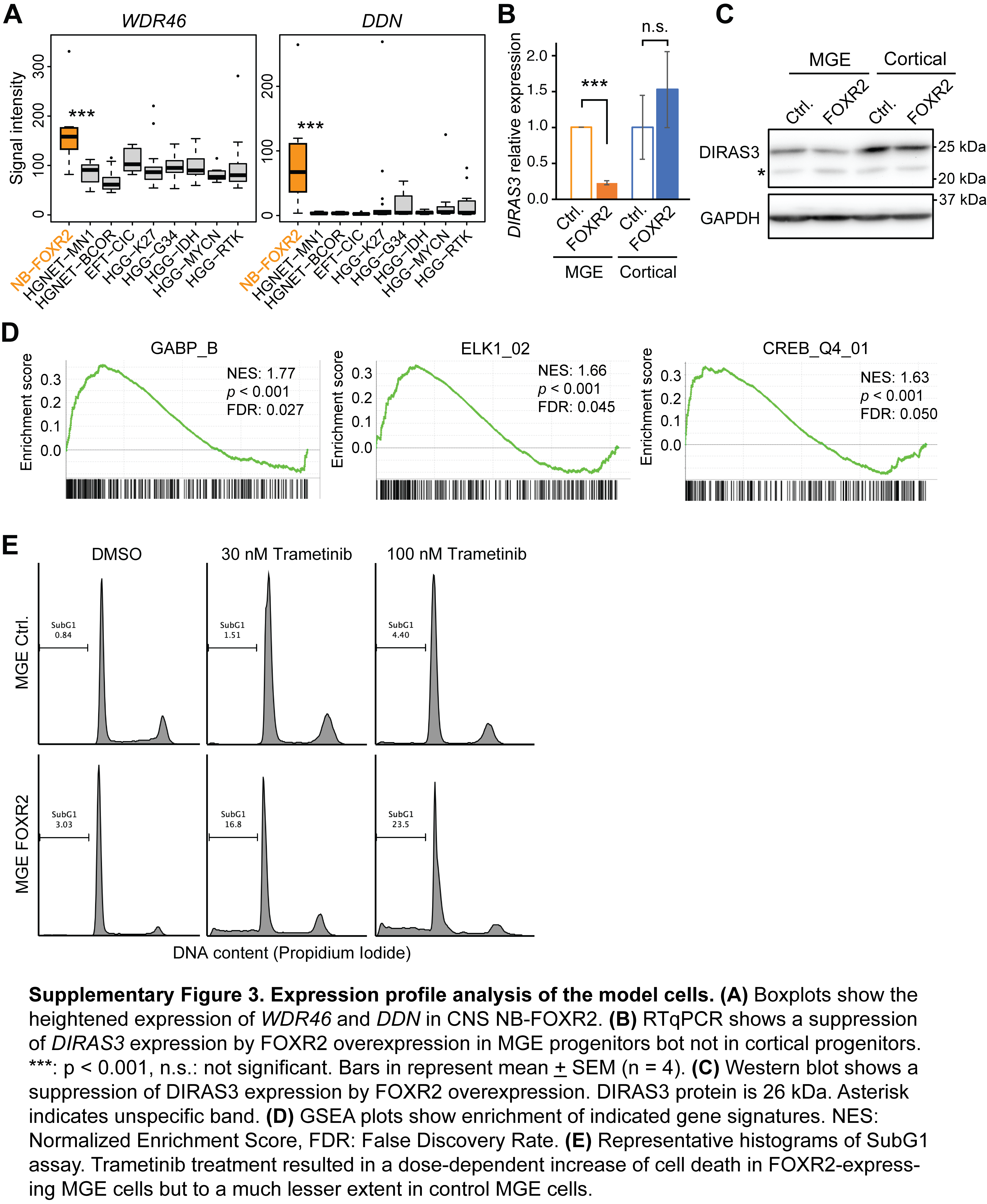

Supplement: vdae144_suppl_Supplementary_Data [file vdae144_suppl_supplementary_data.zip › Royston_et_al_FOXR2_FigS3c.tif]
